# Dynamic and Comparative Transcriptome Analyses Reveal Key Factors Contributing to Cadmium Tolerance in Broomcorn Millet

**DOI:** 10.3390/ijms23116148

**Published:** 2022-05-30

**Authors:** Jiajia Liu, Dazhong Zhang, Yuanbo Zhang, Hao Zhou, Pengliang Chen, Yuhao Yuan, Qinghua Yang, Lin Zhao, Baili Feng

**Affiliations:** 1 State Key Laboratory of Crop Stress Biology in Arid Areas, College of Agronomy, Northwest A & F University, Xianyang 712100, China; ljjzl2014@163.com (J.L.); zdz1727697945@163.com (D.Z.); yuanbo@nwafu.edu.cn (Y.Z.); nongdahaozhou@163.com (H.Z.); cpl2020@126.com (P.C.); yuanyuhao@nwafu.edu.cn (Y.Y.); qinghuayang@nwafu.edu.cn (Q.Y.); 2 Shaanxi Provincial Research Academy of Environmental Sciences, Xi’an 710061, China

**Keywords:** cadmium stress, cell wall, subcellular distribution, transcriptome, transporter gene

## Abstract

Broomcorn millet (*Panicum miliaceum* L.) has great potential in Cd phytoextraction, but its mechanisms are largely unknown. Two contrasting broomcorn millet varieties, ‘Ningmi6’ (Cd-sensitive variety) and ‘4452’ (Cd-tolerant variety), were investigated through morphological, physiological, and transcriptomic analyses to determine the factors responsible for their differential Cd tolerance and translocation. The Cd-tolerant variety can accumulate more Cd, and its cell wall and vacuole component Cd proportions were higher compared with the Cd-sensitive variety. Under Cd stress, the glutathione content and peroxidase activity of the Cd-tolerant variety were significantly higher than those of the Cd-sensitive variety. Additionally, weighted gene co-expression network analysis (WGCNA) revealed hub modules that were associated with Cd stress and/or variety. Notably, genes involved in these hub modules were significantly enriched for roles in glutathione metabolism, phenylpropanoid biosynthesis, ABC transport, and metal ion transport process. These results suggested that regulation of genes associated with cell wall precipitation and vacuole compartmentalization may increase Cd tolerance and reduce Cd translocation in the Cd-tolerant variety, although it can absorb more Cd. This study provides a foundation for exploring molecular mechanisms of Cd tolerance and transport in broomcorn millet and new insights into improving Cd phytoremediation with this crop through genetic engineering.

## 1. Introduction

Cadmium (Cd) is one of the most toxic environmental pollutants derived from natural sources as well as human activities. Cd has no biological function for plant growth and development, but can profoundly interfere with a series of physiological processes, resulting in significant growth retardation and loss of plant productivity [1,2]. Worse still, Cd can enter the human body through the food chain and cause a variety of diseases, posing a serious threat to human health [3,4]. Therefore, remediation and reasonable utilization of Cd-contaminated soil are urgently needed. Phytoremediation technology is an elegant, economical, environmentally friendly, and publicly accepted in situ bioremediation technique [5]. As one of the phytoremediation techniques, phytoextraction employs metal-accumulating plants, such as white cabbage (*Brassica oleracea*) [6], hemp (*Cannabis sativa*) [7], and sweet sorghum (*Sorghum bicolor*) [8], to translocate heavy metals from the soil and accumulate them in harvestable parts [6]. However, its application still faces challenges because it is heavily dependent on soil Cd concentrations and the abilities of available hyperaccumulators [9,10].

Broomcorn millet (*Panicum miliaceum* L.) is probably one of the oldest cereal, industry, and bioenergy crops widely cultivated in Asia, Europe, and the USA [11,12,13]. Broomcorn millet has been referred to as both a “smart food” and “pioneer crop” because it exhibits high tolerance to various biotic and abiotic stresses and it is critical for enduring food security in the face of challenges such as the present COVID-19 pandemic [13,14,15]. Furthermore, a rich germplasm resource has been developed for broomcorn millet [16], and our previous research has revealed that some broomcorn millet varieties are hyperaccumulators with potential for Cd phytoextraction [17]. However, mechanisms of Cd tolerance, absorption, and transport in broomcorn millet are still unknown.

As the first organ to encounter Cd in the growth substratum, roots largely regulate Cd tolerance, uptake, and transport in plants [18]. Roots have evolved diverse defense mechanisms to cope with heavy metals, such as extrusion, precipitation of Cd in the cell wall, activation of antioxidant defense systems, vacuolar compartmentalization, chelation, and transcriptional regulation [7,19,20]. Moreover, roots have vital functions in Cd uptake and xylem loading, which are the foremost processes of Cd transport from soil to the aboveground parts of plants [18,21]. These Cd uptake and translocation processes require the involvement of transporters [22], such as zinc-regulated transporter/iron-regulated transporter-like protein (ZIP) transporters, natural resistance-associated macrophage protein (Nramp) transporters, the heavy metal ATPase (HMA) transporters, and ABC transporters. For newly discovered hyperaccumulators, it is of great significance to further reveal their mechanisms of Cd tolerance and accumulation by analyzing the key processes and genes in their responses to Cd stress. In recent years, RNA sequencing (RNA-Seq) techniques have been extensively implemented to elucidate different Cd-stress-response mechanisms in various plants [7,8,23]. Nevertheless, the mechanism underlying the response of broomcorn millet to Cd stress remains obscure.

In the present study, the phenotypic, physiological, microstructural, and transcriptional responses to Cd stress of two broomcorn millet varieties with contrasting Cd tolerances were investigated. This study aimed to identify key processes and genes that are responsible for Cd tolerance and translocation. These data could be helpful to uncover the molecular mechanisms related to Cd tolerance and accumulation in broomcorn millet, which provides a foundation for further improving the efficiency of Cd phytoextraction.

## 2. Results

### 2.1. Broomcorn Millet Varieties Exhibited Great Differences in Response to Cd Stress

**Figure 1:**
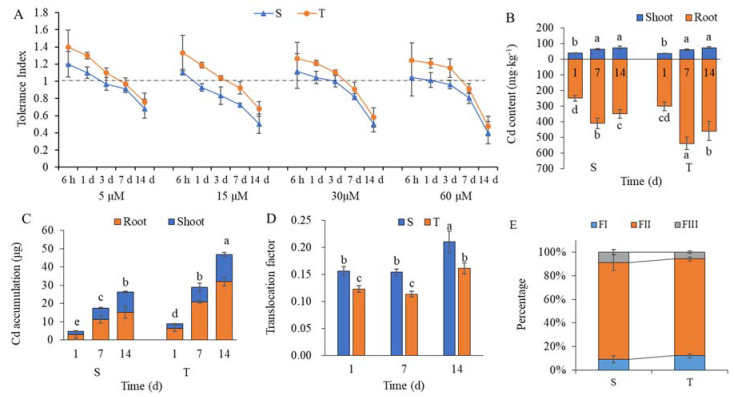
Effect of Cd stress on Cd tolerance index (**A**), concentration (**B**), accumulation (**C**), translocation factor (**D**), and subcellular distribution (**E**) of broomcorn millet varieties S (Cd-sensitive) and T (Cd-tolerant). The results shown in the graphs are means ± SE (*n* = 3). The timepoints at 6 h, 1 day, 3 days, 7 days, and 14 days represent the range of Cd stress time durations, while 5 μM, 15 μM, 30 μM, and 60 μmol/L represent the range of Cd stress concentrations. Different lowercase letters indicate significant differences at a *p* < 0.05 threshold among different Cd treatments and varieties.

To compare the effects of Cd on the two broomcorn millet varieties, growth parameters were detected under Cd treatments with different stress concentrations (0, 5, 15, 30, and 60 μmol/L Cd) and at different time points (0, 6 h, 1 day, 3 days, 7 days, and 14 days). Cd stress concentration (C) and stress time duration (ST), as well as their interaction, had significant effects on shoot length, root length, shoot weight, and root weight (Table S1). Moreover, variety and the interaction of variety and Cd stress concentration had significant effects on root length and weight. Overall, Cd inhibited the growth of broomcorn millet with the extension of stress time, and the inhibition grew more severe as Cd concentration increased. In addition, the tolerance index of the Cd-tolerant variety was higher than that of the Cd-sensitive variety under Cd stress conditions (Figure 1A). The root Cd concentrations were significantly higher in the Cd-tolerant variety relative to the Cd-sensitive variety at 7 and 14 days, while shoot Cd concentrations showed no significant difference between the two varieties at the same time points of Cd stress (Figure 1B). Notably, Cd accumulation per plant was significantly higher in the Cd-tolerant variety than in the Cd-sensitive variety at the same time points of Cd stress (Figure 1C). However, Cd translocation factors were significantly higher in the Cd-sensitive variety relative to the Cd-tolerant variety (Figure 1D). In addition, most Cd was present in the soluble and cell wall fractions in both varieties, but the percentage of soluble and cell wall Cd was higher in the Cd-tolerant variety than in the Cd-sensitive variety (Figure 1E).

### 2.2. Microstructural Difference of the Two Broomcorn Millet Varieties under Cd Stress

**Figure 2:**
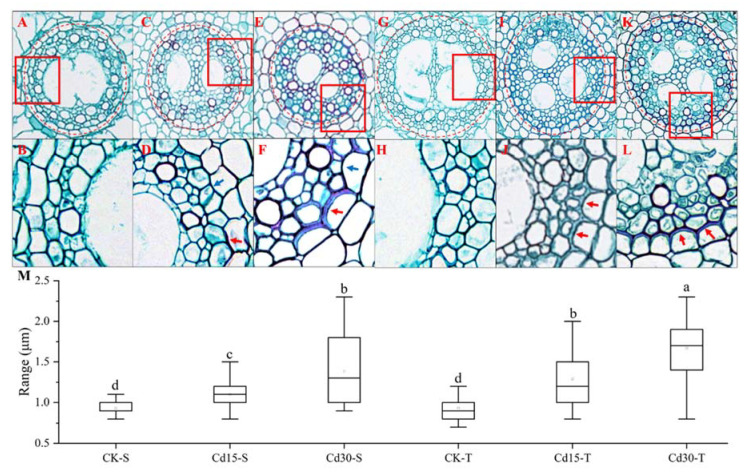
Effect of Cd stress on root microphotographs and cell wall thickness of broomcorn millet. (**A**,**C**,**E**) Optical micrographs of Cd-sensitive variety under control conditions (CK-S), 15 μmol/L Cd stress treatment (Cd15-S), and 30 μmol/L Cd stress treatment (Cd30-S), respectively. (**G**,**I**,**K**) Optical micrographs of Cd-tolerant variety under control conditions (CK-T), 15 μmol/L Cd stress treatment (Cd15-T), and 30 μmol/L Cd stress treatment (Cd30-T), respectively. Partial magnified images of (**A**,**C**,**E**,**G**,**I**,**K**) (red boxed areas) are shown as (**B**,**D**,**F**,**H**,**J**,**L**), respectively. (**M**) Statistics of cell wall thickness of the cells labeled by red circles. Cd-sensitive and Cd-tolerant cultivars. The red arrows represent obvious thickness of the cell wall, while blue arrows represent not obvious thickness of the cell wall. Different lowercase letters indicate significant differences in mean values at a *p* < 0.05 threshold among different Cd treatments and varieties.

Cd treatment caused anatomical alterations of the root with endodermal cell wall thickening (Figure 2). As Cd concentration increased, thickenings of endodermal cell walls became more obvious, with the thickening in the Cd-tolerant variety (Figure 2I–L) being more obvious than that in the Cd-sensitive variety (Figure 2B–E). The thicknesses of cell walls were from 0.8 to 1.1 μm, from 0.8 to 1.4 μm, and from 0.9 to 2.2 μm in the Cd-sensitive variety under control, 15, and 30 μmol/L Cd treatments, respectively; they were from 0.7 to 1.2 μm, from 0.8 to 2 μm, and from 0.8 to 2.7 μm in the Cd-tolerant variety under control, 15, and 30 μmol/L Cd treatments, respectively (Figure 2M). Compared with control, the mean value of cell wall thickness was significantly higher in both varieties under Cd stress. Moreover, compared with the Cd-sensitive variety, the mean cell wall thickness was significantly higher in the Cd-tolerant variety under the same Cd stress conditions, indicating that the Cd-tolerant variety may be more efficient in cell wall fixation of Cd.

### 2.3. Root Physiological Differences in Response to Cd Stress

**Figure 3:**
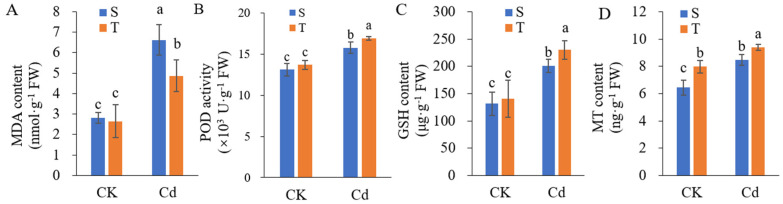
The malondialdehyde (MDA) content (**A**), peroxidase (POD) activity (**B**), glutathione (GSH) content (**C**), and metallothionein (MT) content (**D**) in the Cd-sensitive variety (S) and Cd-tolerant variety (T) under control (CK) and Cd stress conditions. The values shown are means ± SE (*n* = 3). Different lowercase letters above each bar represent significant differences among treatments and varieties (*p* < 0.05).

The MDA content was increased significantly in both varieties under Cd stress, and the MDA content in the Cd-sensitive variety was significantly higher than that in the Cd-tolerant variety (Figure 3A). The activities of POD (Figure 3B) increased under Cd stress in both varieties; however, the activity levels were significantly higher in the Cd-tolerant variety than in the Cd-sensitive variety. Compared with control conditions, GSH (Figure 3C) and MT (Figure 3D) contents also increased significantly in both varieties under Cd stress. The GSH content was significantly higher in the Cd-tolerant variety than in the Cd-sensitive variety under Cd stress, while MT content was remarkably higher in the Cd-tolerant variety than in the Cd-sensitive variety under both control and Cd stress conditions. Therefore, the Cd-tolerant variety exhibited more efficient antioxidation and chelation, likely contributing to its higher Cd tolerance.

### 2.4. Overview of Transcriptome Sequencing Results

A total of 36 RNA libraries were created with sequencing errors of less than 0.025%. The average Q30 and GC contents were 95.8% and 53.8%, respectively. Mapping of reads with the reference genome showed alignments of 81.88–95.78% (Table S2). Moreover, correlation coefficients between the replicates were generally higher than 0.90 (Figure S1A), and the replicates with lower correlation coefficients were removed from subsequent analyses. Moreover, RT-qPCR analysis showed that expression patterns from 9 of 10 genes were significantly consistent with those of RNA-Seq data (Figure S1B), indicating that the RNA-Seq data were highly accurate. Further, the PCA results revealed substantial differences between the two varieties and among the different Cd stress stages (Figure S1C). Overall, these findings indicate the fine quality of the sequencing results.

### 2.5. Differentially Expressed Genes and Their Dynamic Responses to Cd Stress

**Figure 4:**
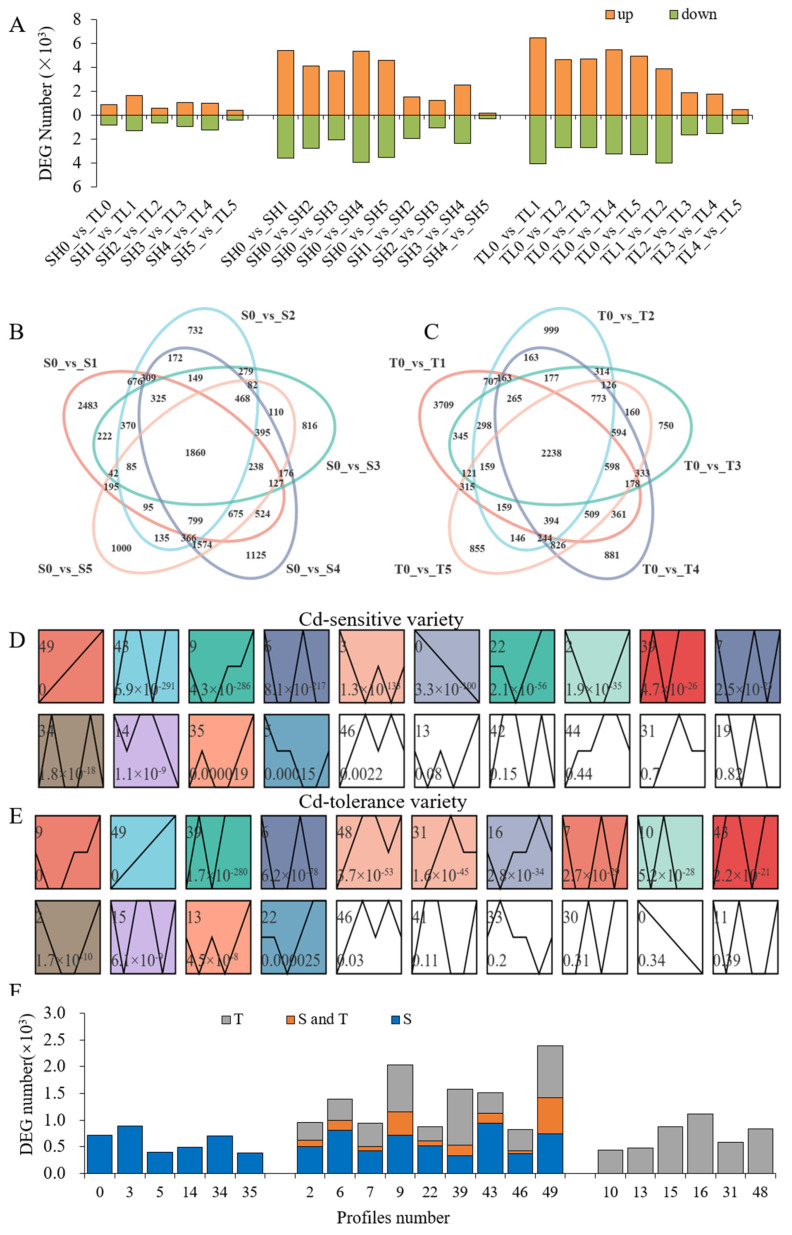
The number of differentially expressed genes (DEGs) between the two varieties and between different Cd stress time points (**A**). Venn analysis of DEGs between control and Cd stress conditions in the Cd-sensitive variety (**B**) and Cd-tolerant variety (**C**). Short Time-Series Expression Miner (STEM) analysis of DEGs in the Cd-sensitive variety (**D**) and Cd-tolerant variety (**E**). Summary of DEGs in each profile with a significant change trend (**F**). S0, S1, S2, S3, S4, and S5 represent Cd-sensitive variety under control, 6 h, 1-day, 3-day, 7-day, and 14-day Cd treatments, respectively; T0, T1, T2, T3, T4, and T5 represent Cd-tolerant variety under control, 6 h, 1-day, 3-day, 7-day, and 14-day Cd treatments, respectively.

Compared with the Cd-sensitive variety, there were 1704 genes expressed differentially in the Cd-tolerant variety under control conditions; this value reached 2927 after 6 h of Cd stress, and ultimately, this value decreased to 806 after 14 days of Cd stress (Figure 4A). The trends in DEGs were similar under different Cd stress time points compared with the control conditions for the two varieties; however, there were more DEGs, especially upregulated DEGs, between the Cd stress and control treatments in the Cd-tolerant variety than Cd-sensitive variety (Figure 4A). Totals of 1860 (Figure 4B) and 2238 (Figure 4C) DEGs were common between control conditions and different Cd stress time points in the Cd-sensitive variety and Cd-tolerant variety, respectively. With prolonged Cd stress, DEGs in the Cd-sensitive variety (Figure 4D) and Cd-tolerant variety (Figure 4E) showed 15 significant changing tendencies, and 9 of them existed in both varieties. However, the DEGs were not identical, although the expression trends were consistent (Figure 4F). These results indicated that Cd stress induced strong changes in transcriptional levels; these transcriptional responses were complex and showed obvious differences between the two varieties under prolonged Cd stress.

### 2.6. Identification of Hub Modules through WGCNA

**Figure 5:**
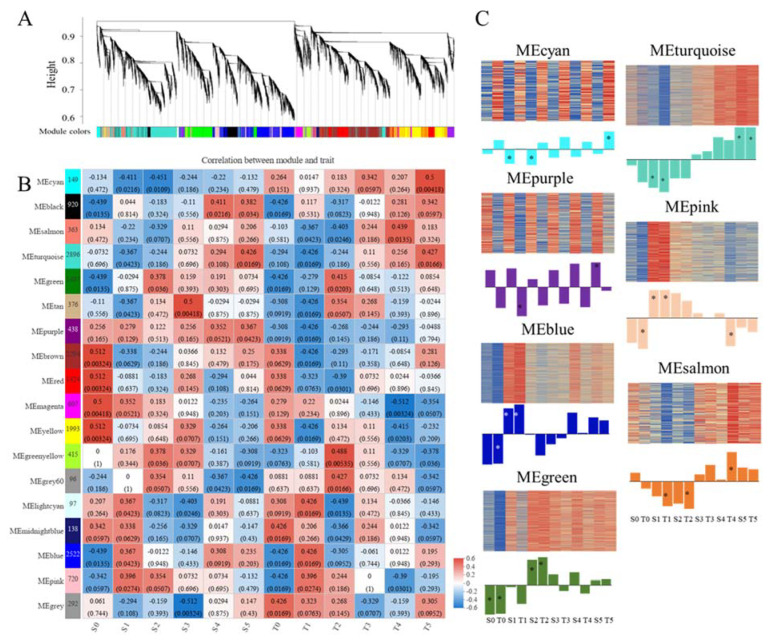
Hierarchical clustering tree of DEGs based on co-expression network analysis in the Cd-sensitive and Cd-tolerant varieties (**A**), eigengene–trait correlation analysis (**B**), and expression patterns of the co-expressed genes in hub modules (**C**). Numbers on the heatmap represent correlation coefficients, and numbers less than 0.05 in parentheses indicate significant correlations. * represent significant correlations.

In order to reveal key factors associated with Cd tolerance and translocation in broomcorn millet, WGCNA analysis was used to analyze these DEGs (a total of 17,237 after removing very low expression and/or low variable coefficient values) between the two varieties, and between control and Cd stress. Through WGCNA, 18 co-expression modules were ultimately identified, with the number of DEGs in each module ranging from 96 to 2896 (Figure 5A,B). Moreover, eigengene–trait correlation analysis showed seven co-expression modules (MEcyan, MEpurple, MEblue, MEgreen, MEturquoise, MEpink, and MEsalmon) with relatively higher correlations were considered as hub modules (Figure 5B). Gene expression patterns of these hub modules were further analyzed (Figure 5C), and results showed that DEGs in the MEcyan and MEpurple may be related to various characteristics. DEGs in MEblue and MEgreen may primarily be involved in short-term responses to Cd stress. However, the DEGs in MEturquoise may primarily be involved in both short-term and long-term responses to Cd stress. DEGs in MEpink may be involved in short-term responses to Cd stress in both varieties, but only associated with long-term responses of the Cd-tolerant variety. Moreover, DEGs in MEsalmon may participate in the detoxification response to Cd stress in the Cd-tolerant variety.

### 2.7. GO and KEGG Enrichment Analysis of DEGs in Hub Modules

GO enrichment analysis of each module highlighted key biological processes represented by a set of co-expressed genes (Table S3). GO analyses indicated that DEGs in MEgreen were significantly enriched for GO terms associated with cell wall metabolism. In addition, GO analyses showed that DEGs were significantly enriched for GO terms related to glutathione transferase activity, cinnamic acid biosynthetic process, and plasma membrane in MEblue and hydrogen peroxide catabolic process, oxidoreductase activity, and detoxification in MEpink. These DEGs were significantly enriched for GO terms that included transition metal ion transport, zinc ion transport, zinc ion transmembrane transporter activity, and metal ion homeostasis. The functions of DEGs in MEsalmon were significantly enriched for GO terms related to plant-type cell wall organization and oxidoreductase activity.

**Figure 6:**
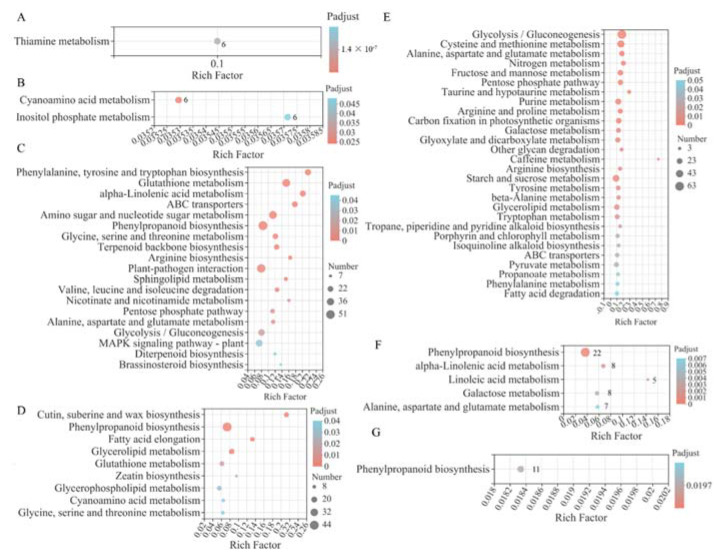
KEGG enrichment analyses of DEGs involved in MEcyan (**A**), MEpurple (**B**), MEblue (**C**), MEgreen (**D**), MEturquoise (**E**), MEpink (**F**), and MEsalmon (**G**). The numbers beside the bubbles in (**A**,**B**,**F**,**G**) represent the numbers of genes.

KEGG enrichment analyses (Figure 6) suggested that metabolisms related to carbohydrates (including fructose and mannose metabolism, galactose metabolism, and glycolysis/gluconeogenesis), lipids (including fatty acid elongation and glycerolipid metabolism), and amino acids (including glycine, serine, and threonine metabolism; alanine, aspartate, and glutamate metabolism; and cysteine and methionine metabolism) were significantly enriched in these hub modules. Notably, the phenylpropanoid biosynthesis pathway was significantly enriched in four modules, namely MEblue (Figure 6C), MEgreen (Figure 6D), MEpink (Figure 6F), and MEsalmon (Figure 6G). Additionally, DEGs in MEblue (Figure 6C) and MEgreen (Figure 6D) were significantly enriched for involvement in the glutathione metabolism pathway; DEGs in MEblue (Figure 6C) and MEturquoise (Figure 6E) were significantly enriched in the ABC transporter pathway. These findings indicated that Cd stress induced obvious changes in metabolism pathways, and phenylpropanoid biosynthesis, glutathione metabolism, and the ABC transporter pathway may play a vital role in Cd tolerance and transport.

### 2.8. DEGs Involved in Phenylpropanoid Biosynthesis and GSH Metabolism Pathway

**Figure 7:**
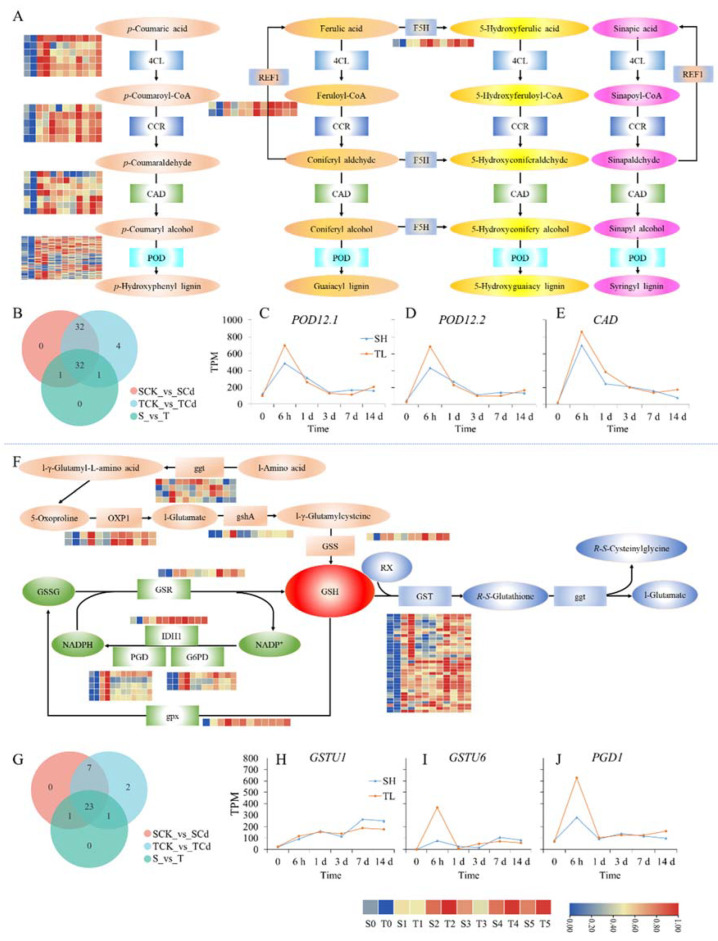
Expression pattern analysis of differentially expressed genes (DEGs) involved in phenylpropanoid biosynthesis pathway (**A**) and glutathione metabolism pathway (**F**); Venn diagrams of PODs (**B**) and GSTs (**G**); and expression analysis of *POD12.1* (**C**), *POD12.2* (**D**), *CAD* (**E**), *GSTU1* (**H**), *GSTU6* (**I**), and *PGD1* (**J**). SCK_vs._SCd, TCK_vs._TCd, and S_vs._T represent DEGs between control and Cd treatments in the Cd-sensitive variety, between control and Cd treatments in the Cd-tolerant variety, and between the two varieties, respectively. REF1, 4CL, CCR, CAD, F5H, ggt, OXP1, gshA, GSS, GSR, IDH1, PGD, G6PD, gpx, and GST represent coniferyl-aldehyde de-hydrogenase, 4-coumarate-CoA ligase, cinnamoyl-CoA reductase, cinnamyl-alcohol dehydrogenase, ferulate-5-hydroxylase, gamma-glutamyltranspeptidase, 5-oxoprolinase (ATP-hydrolyzing), glutamate-cysteine ligase, glutathione synthase, glutathione reductase (NADPH), isocitrate dehydrogenase, 6-phosphogluconate dehydrogenase, glucose-6-phosphate 1-dehydrogenase, glutathione peroxidase, and glutathione S-transferase, respectively.

A total of 128 DEGs from hub modules were enriched in the phenylpropanoid synthesis pathway. About three-quarters of these DEGs were involved in the biosynthesis process from p-coumaric acid, ferulic acid, 5-hydroxyferulic acid, and sinapic acid to p-hydroxyphenyl lignin, guaiacyl lignin, 5-hydroxyguaiacyl lignin, and syringyl lignin, respectively (Figure 7A). Notably, more than half of the DEGs were peroxidase (POD) genes. Venn analysis showed that 34 POD genes were induced by Cd stress and significantly differentially expressed between the two varieties and 5 POD genes were only induced by Cd stress in the Cd-tolerant variety (Figure 7B). Moreover, among these POD genes, *POD12.1* (Figure 7C) and *POD12.1* (Figure 7D) were highly expressed and induced by short-term Cd stress. Meanwhile, their expression levels were higher in the Cd-tolerant variety than in the Cd-sensitive variety under Cd stress for 6 h. Moreover, the *CAD* gene encoding cinnamyl alcohol dehydrogenase was also highly expressed and significantly induced by short-term Cd stress, and its expression level was higher in the Cd-tolerant variety than in the Cd-sensitive variety after 6 h, 3 days, and 14 days of Cd stress (Figure 7E).

The catalytic enzyme genes involved in GSH biosynthesis, the GSH-GSSG cycle, and GSH complex synthesis were induced by Cd stress and differentially expressed between the two varieties (Figure 7F). Among the identified DEGs, the number of DEGs encoding glutathione S-transferase (GST) was the greatest, at 34. Venn analysis showed that 30 GST genes were induced by Cd stress in both varieties, among which 7 GST genes were significantly differentially expressed between the two cultivars (Figure 7G). Moreover, among these GST genes, *GSTU1* and *GSTU6* were highly expressed. The expression level of *GSTU1* was maintained at a high level with the prolongation of Cd stress duration, and it was higher in the Cd-sensitive variety than in the Cd-tolerant variety under Cd stress for 7 and 14 days (Figure 7H). However, *GSTU6* was induced by short-term Cd stress, and its expression level was higher in the Cd-tolerant variety than in the Cd-sensitive variety under Cd stress for 6 h (Figure 7I). In addition, the *PGD1* gene encoding 6-phosphogluconate dehydrogenase involved in the GSH-GSSG cycle was also highly expressed and significantly induced by short-term Cd stress, and its expression level was higher in the Cd-tolerant variety under Cd stress for 6 h and 14 days (Figure 7J).

### 2.9. Transporter Genes Involved in Cd Uptake and Translocation

**Figure 8:**
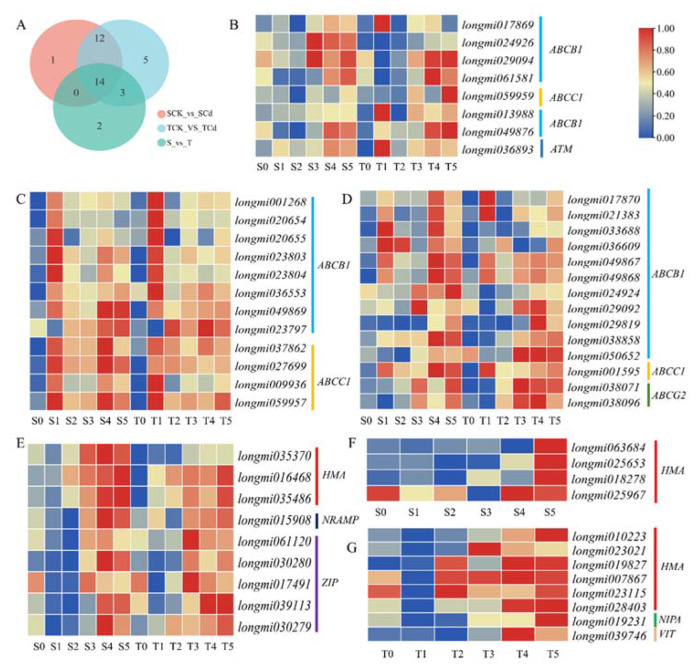
Venn diagram of differentially expressed genes (DEGs) involved in ABC transport pathway (**A**), expression pattern heatmaps of (**B**) DEGs induced by Cd stress in the Cd-tolerant variety, (**C**) DEGs induced by Cd stress in the two varieties, (**D**) DEGs induced by Cd stress and differently expressed in the two varieties, (**E**) DEGs induced by Cd stress and differently expressed in the two varieties, (**F**) DEGs induced by Cd stress in the Cd-sensitive variety, and (**G**) DEGs induced by Cd stress in Cd-tolerant variety. SCK_vs._SCd, TCK_vs._TCd, and S_vs._T represent DEGs between control and Cd treatments in the Cd-sensitive variety, between control and Cd treatments in the Cd-tolerant variety, and between the two varieties, respectively.

In the significantly enriched ABC transporter pathway, a total of 37 ABC transporter genes (including 21 ABCB1 genes, 2 ABCB9 genes, 11 ABCC1 genes, 2 ABCG2 genes, and 1 ATM gene) were induced by Cd stress or differentially expressed between the two broomcorn millet varieties (Figure 8). Among these ABC transporter genes, eight genes were induced by Cd stress in the Cd-tolerant variety and three genes were differentially expressed between the two varieties (Figure 8A,B). A total of 26 genes were induced by Cd stress in both varieties (Figure 6A,C,D), while 14 of them were differentially expressed between the two varieties (Figure 8D). These ABC transporter genes, especially the eight genes that were induced by Cd stress only in the Cd-tolerant variety, may contribute to the high Cd tolerance and accumulation of the Cd-tolerant variety. In addition, transporter genes related to metal ion transport were discovered; nine genes (including four HMA, one NARMP, and five ZIP genes) were induced by Cd stress in both varieties (Figure 8E), while four HMA genes were only induced by long-term Cd stress in the Cd-sensitive variety (Figure 8F). Notably, eight genes, including six HMA genes, one magnesium transporter gene (NIPA), and one vacuolar iron transporter gene (VIT) were induced by Cd stress in the Cd-tolerant variety (Figure 8G). These transporter genes may contribute to the difference in Cd accumulation and translocation between the two varieties.

## 3. Discussion

According to the data of phenotype and Cd concentration and accumulation under different Cd treatment concentrations and processing time, ‘Ningmi6’ was considered to be a Cd-sensitive variety with lower Cd accumulation, while ‘4452’ was a Cd-tolerant variety with higher Cd accumulation. Physiological, cytological, and dynamic transcriptome analyses were used to explore mechanisms of Cd tolerance and accumulation in broomcorn millet. 

In plants growing on Cd-contaminated soils, the root cell wall is the first component of plants to contact Cd, and it is thus the first barrier preventing metals from entering into cells [20]. Cellulose, hemicellulose, pectin, and glycoprotein are the main components of plant cell walls, and these polysaccharides and proteins contain many chemical functional groups, such as –COOH, −OH, and –SH groups, which are able to effectively bind divalent and trivalent metal cations [24]. Previous studies have demonstrated that under Cd stress, the thickness and components of cell walls change to immobilize more Cd for detoxification of the whole plant [18,25,26]. Under Cd stress, root cell wall thickening and cell wall Cd concentration were more obvious in the Cd-tolerant variety than in the Cd-sensitive variety in the current study, which may be one of the reasons that the Cd-tolerant variety had higher Cd tolerance and lower Cd translocation from root to shoot. Moreover, many DEGs were found to be linked to cell wall biogenesis and modification, cell wall macromolecule (pectin, cellulose, lignin, and suberin) catabolic processes, and phenylpropanoid biosynthesis pathways under Cd stress, and these results were consistent with the results found for sweet sorghum [8]. These findings indicated that cell wall metabolism was obviously induced by Cd stress and the cell wall was beneficial to Cd fixation, reducing Cd toxicity and translocation and thus improving Cd tolerance in broomcorn millet.

Lignin is also an important component of the cell wall and plays a critical role in plant resistance to stress. The phenylpropanoid pathway contains a series of intricate branching biochemical reactions and can generate numerous secondary metabolites, which serve as metabolite sources for lignin biosynthesis [8,27]. Thus, upregulated phenylpropanoid biosynthesis could facilitate the fixation of Cd on the lignin of the cell wall [28]. Consistent with the results of Wang et al. [29], many DEGs were associated with the phenylpropanoid biosynthesis pathway in the current study. Therefore, the DEGs of these hub modules were further analyzed, and most of these DEGs were related to lignin biosynthesis. Notably, more than half of DEGs involved in POD and two highly expressed POD-encoding genes (*POD12.1* and *POD 12.2*) were induced by short-term Cd stress with higher expression in the Cd-tolerant variety. Besides its antioxidant properties, POD can also catalyze the synthesis of lignin, induce cell wall stiffening, and affect the transportation of heavy metals [28,30,31]. According to the physiological data, POD activity was significantly increased under Cd stress and showed higher activity in the Cd-tolerant variety, suggesting POD genes such as *POD12.1* and *POD 12.2* may be responsible for Cd tolerance and low translocation in Cd-tolerant variety. Moreover, CAD, an enzyme catalyzing the last step of lignin synthesis, was reported to enhance lignin synthesis in *Zanthoxylum bungeanum* under cold stress [32]. In the current study, the *CAD* gene was also significantly induced by Cd stress, and its expression was higher in Cd-tolerant variety than in the Cd-sensitive variety after Cd stress for 6 h, 1 day, and 14 days. These findings indicate that an enhancement of lignin synthesis may be an important strategy for resisting Cd stress in broomcorn millet, and *POD12.1*, *POD 12.2*, and *CAD* may be key genes involved in this process.

Glutathione (GSH) metabolism was one of the key pathways involved in the response of broomcorn millet to Cd stress, which was consistent with previous studies on *Solanum nigrum* [29]. GSH, a thiol-containing compound, is a bioactive tripeptide involved in many aspects of plant metabolism. GSH is known to be involved in peroxide metabolism and redox signaling, and thus, it can regulate the redox state of cells. However, GSH is also the precursor of phytochelatins (PCs), which are widely accepted as having a major role in plant detoxification and tolerance to heavy metal stress [33,34]. GSH has been reported to enhance Cd tolerance and affect Cd transport and accumulation characteristics in plants. For example, GSH can directly or indirectly alleviate Cd toxicity in *Zea mays* by lessening reactive oxygen species damage, maintaining cell redox homeostasis, improving cell ultrastructure, ameliorating Cd-induced photosynthesis inhibition, and activating a series of TF and Cd-tolerance-related genes, as well as sequestering more Cd^2+^ ions into vacuoles and reducing root-to-shoot Cd transport [35]. In addition, research on *Arabidopsis* has demonstrated that enhancing endogenous and exogenous GSH can decrease the Cd translocation ratio [36]. In this study, GSH content was significantly increased under Cd stress, and the GSH content was significantly higher in Cd-tolerant variety than in the Cd-sensitive variety. Moreover, compared with the Cd-sensitive variety, vacuole Cd content was higher in the Cd-tolerant variety, indicating that GSH was involved in the Cd chelation of broomcorn millet.

Notably, a number of genes encoding GST, which are thus involved in the GSH metabolism pathway, were significantly differentially expressed under Cd stress and between the two broomcorn millet varieties. GSTs comprise a family of multifunctional proteins that have important roles in defending cells against potentially toxic compounds by catalyzing the conjugation of reactive metabolites to GSH [37,38]. Additionally, based on transcriptome data, Wu et al. [38] found a number of GST genes that responded to Cd stress in pak choi (*Brassica chinensis*) and furthermore demonstrated that one of them, *BcGSTU11*, increased the Cd tolerance of yeast [37]. The current results revealed that *GSTU1* (highly expressed in the Cd-sensitive variety under long-term Cd stress) and *GSTU6* (highly expressed in Cd-tolerant variety under short-term Cd stress) may negatively and positively regulate Cd tolerance in broomcorn millet, respectively, suggesting these two GST genes may have different functions in response to Cd stress and merit further study.

Unlike essential plant nutrients, there is no specific transporter responsible for Cd uptake and transport. Thus, it has been speculated that Cd is transported into cells via the transporters responsible for the transport of essential divalent cations [19]. Given our previous study showing that Cd and Zn concentrations are positively correlated in broomcorn millet under Cd stress [17], transporters associated with uptake and transport of Zn^2+^, such as ZIP family transporter, may play key roles in Cd uptake and transport in broomcorn millet. Based on transcriptome data, ZIP transporter family genes were induced by Cd stress and expressed differentially between the two broomcorn millet varieties, and these results were consistent with research on hemp [7], suggesting that ZIP transporters might regulate Cd uptake and influence Cd resistance. Moreover, functions of some genes in these transporter families have been shown to be involved in Cd uptake, translocation, and retention in plants. For example, some of these proteins are important phytochelatin transporters, such as AtABCC1, AtABCC2, and AtABCC3, which can transport PC–Cd complexes into vacuoles for sequestration and thereby reduce Cd translocation to the shoots [39,40]. In the current study, an *ABCC1* gene (*longmi059959*) was only induced by Cd stress in the Cd-tolerant variety, suggesting that it may be an important gene responsible for vacuole sequestration and lower Cd translocation in the Cd-tolerant variety. For phytoextraction, the creation of hyperaccumulators by transgenic or gene editing techniques targeting these transporters may be a powerful means to achieve improved remediation of contaminated soils [22].

## 4. Materials and Methods

### 4.1. Transporter Genes Involved in Cd Uptake and Translocation

Two broomcorn millet (*Panicum miliaceum* L.) cultivars with contrasting Cd tolerances and translocation factors were used as materials. ‘Ningmi6’ is a Cd-sensitive variety, while ‘4452’ is a Cd-tolerant variety [17].

The seedlings were cultured hydroponically. Seeds of similar size and appearance were sterilized with 0.1% mercuric chloride (HgCl_2_) for 10 min, rinsed thrice with sterile water, and placed on moistened filter paper in Petri dishes for 3 days at 25 °C in the dark. Seedling culture was carried out by referring to the method of Liu et al. [17]. At the four-leaf stage, the seedlings were treated with nutrient solution containing 0 (control, CK), 5, 15, 30, or 60 μmol/L Cd (as CdCl_2_·2.5H_2_O) with three replicates for each treatment. The doses of Cd were selected based on the work of Zhang et al. [18]. The seedlings were harvested at 0 h, 6 h, 1 day, 3 days, 7 days, and 14 days after initiation of Cd treatment. A portion of these samples was stored at 4 ℃, while another portion of these samples was frozen in liquid nitrogen and stored in a −80 ℃ refrigerator for the following experiments.

### 4.2. Determination of Phenotypic and Cd-Related Traits

Shoot length (SL), maximum root length (MRL), shoot weight (SW), and root weight (RW) of the seedlings were measured at 0 h, 6 h, 1 day, 3 days, 7 days, and 14 days after initiation of the 0, 5, 15, 30, and 60 μmol/L Cd treatments. The Cd tolerance index was defined as the average of the relative SL, MRL, SW, and RW values, in which the relative SL, MRL, SW, and RW values were the ratio of the values of these traits under each Cd stress level to their values under control conditions at each corresponding time point. 

The Cd concentration (CdC, mg·kg^−1^ dry weight) was determined using the method described by Liu et al. [17]. The Cd certified reference material (GSB 04-1721-2004) was used as the standard for CdC determination. Additionally, shoot and root CdC measurements were determined at 1 day, 7 days, and 14 days after initiation of the 15 μmol/L Cd treatment. The Cd translocation factor was recorded as the ratio of shoot CdC to root CdC. In addition, the root samples (stored at −80 °C) treated with 15 μmol/L Cd stress for 14 days were used to determine the Cd subcellular distribution according to the method described by Yang et al. [41]. The cells were separated into three fractions: cell wall fraction (FI), soluble (mainly vacuole) fraction (FII), and organelle fraction (FIII). Then, CdC measurements were determined for each fraction.

### 4.3. Determination of Physiological Parameters

Root samples, collected from plants grown under 15 μmol/L Cd stress for 14 days and from control plants at the same stage, were used to determine the following physiological parameters. The contents of malondialdehyde (MDA), peroxidase (POD), reductive glutathione (GSH), and metallothionein (MT) were determined using test kits according to the manufacturer’s instructions (Solarbio, Beijing, China).

### 4.4. Microstructural Observations of Root Tissu

Fresh root tips (approximately 1 cm in length) were collected from plants under 15 μmol/L Cd stress for 14 days and from control plants at the same stage and then fixed with formaldehyde–acetic acid–ethanol fixative (FAA) for more than 24 h. Paraffin sections of the root tips were prepared as described previously [42] to observe the root cell structure after staining with saffron solid green under an epifluorescence microscope (Carl Zeiss, Oberkochen, Germany). The cell wall thickness was measured by CaseViewer (version 2.0, 3DHISTECH Ltd., Budapest, Hungary) software.

### 4.5. Transcriptome Sequencing and Analysis

Root samples (stored at −80 °C) of the Cd-sensitive variety (S) and Cd-tolerant variety (T) treated with 15 μmol/L Cd stress for 0 h (S0 and T0), 6 h (S1 and T1), 1 day (S2 and T2), 3 days (S3 and T3), 7 days (S4 and T4), and 14 days (S5 and T5) were subjected to transcriptome sequencing. A total of 36 libraries (two varieties × six stages × three replicates) were constructed. The RNA isolation and library construction were performed using the methods described by Chen et al. [43]. Then, the libraries were sequenced by Shanghai Majorbio Biopharm Technology Corporation (Shanghai, China) using the Illumina HiSeq X Ten platform (Illumina, San Diego, CA, USA). The clean reads were mapped to the reference genome of *Panicum miliaceum* (Version GWHAAEZ00000000, http://bigd.big.ac.cn/gwh/Assembly/131/show, accessed on 12 September 2018) [12] using HISAT2 software (http://ccb.jhu.edu/software/hisat2/index.shtml, released on 6 February 2020).

The data were analyzed on the online platform of Majorbio Cloud Platform (www.majorbio.com, accessed on 12 September 2018). Gene expression levels were estimated as transcripts per million reads mapped (TPM). Differentially expressed genes (DEGs) were then analyzed using the DESeq2 package and are presented as log_2_(fold change (FC)) values. An absolute value of |log_2_(FC)| ≥ 1 and *p_adjusted_* < 0.05 were used as the thresholds to judge the significance of gene expression differences. 

Gene function was annotated using BlastX with an E-value cut-off of less than 10^−5^ against Gene Ontology (GO, http://www.geneontology.org, accessed on 12 September 2018) and Kyoto Encyclopedia of Genes and Genomes pathway database (KEGG, http://www.genome.jp/kegg/, accessed on 12 September 2018). GO and KEGG pathway enrichment analyses were conducted to further elucidate the biological functions of DEGs by using the online platform of Majorbio Cloud Platform (www.majorbio.com, accessed on 12 September 2018), and GO terms and KEGG pathways with *p_adjusted_* < 0.05 were considered to be significantly enriched.

In addition to creating Venn diagrams, principal component analysis (PCA), correlation analysis, and the following analysis were also performed using Majorbio Cloud Platform (www.majorbio.com, accessed on 12 September 2018). By analyzing the domain information of the gene transcription products, PlantTFDB 4.0 (http://planttfdb.cbi.pku.edu.cn/, accessed on 12 September 2018) was used to predict transcription factors and identify the families of DEGs. After summarizing the differentially expressed genes, Short Time-Series Expression Miner (STEM) analysis was used to explore the gene expression patterns at multiple time points. Each gene was classified according to its most consistent trend, and the number of time series patterns was set to 50. In addition, weighted gene co-expression network analysis (WGCNA, https://horvath.genetics.ucla.edu/html/CoexpressionNetwork/Rpackages/WGCNA/, released on 26 February 2018) was performed after genes with low expression levels or low variation coefficients were filtered. The Soft Threshold (β power) was set to 6, and all other parameters were set to the platform default values.

### 4.6. Quantitative Real-Time PCR Analysis

The expression levels of 10 DEGs involved in Cd stress identified by the transcriptomic method were verified by using qRT-PCR (quantitative real-time polymerase reaction). The qRT-PCR was performed according to the method decreased by Yuan et al. [44]. The gene *18S* (as actin) was used as the control, and the primer sequences are listed in Table S4.

### 4.7. Statistical Analysis

Phenotypic traits, Cd-related traits, and physiological parameters are presented as means (±SE) of at least three replicates. Data were analyzed with SPSS statistics software (Version 19.0 for Windows; IBM Corp., Armonk, NY, USA). The effects of cultivar, Cd stress concentration, Cd stress time, and their interaction on variables were analyzed by three-way analysis of variance (ANOVA) at *p* < 0.05 and *p* < 0.01 levels of significance. Differences between mean values were determined using the least significant difference (LSD) test at a *p* < 0.05 level. Bar charts and line charts were rendered with Excel 2019 (Microsoft Corp., Redmond, WA, USA). Heat maps of gene expression were drawn with TBtools [45].

## 5. Conclusions

Broomcorn millet has great potential in phytoextraction of Cd-contaminated soils. Revealing the mechanism of Cd tolerance and translocation in broomcorn millet is a critical means to improve Cd phytoextraction efficiency. The current study revealed key genes involved in GSH metabolism, phenylpropanoid biosynthesis, ABC transport, and metal ion transport processes associated with Cd tolerance and transport capacity in broomcorn millet. According to the combined analysis of Cd tolerance index, translocation factor, subcellular distribution, and physiological data, these key genes may promote Cd uptake, cell wall precipitation, Cd chelation, and vacuolar compartmentalization, which may be the reason why the Cd-tolerant variety can absorb more Cd but exhibits less translocation. The comprehensive information presented here will serve as a crucial resource to better understand the mechanisms by which plants cope with Cd stress. These key genes require further study to provide insights that will improve the phytoremediation efficiency of Cd pollution using broomcorn millet.

## Data Availability

Not applicable.

## Supplementary Materials

The following supporting information can be downloaded at: www.mdpi.com/article/10.3390/ijms23116148/s1.
