# Supplementary material for: Dynamic and Comparative Transcriptome Analyses Reveal Key Factors Contributing to Cadmium Tolerance in Broomcorn Millet
Source: Int J Mol Sci. 2022 May 30;23(11):6148. doi: 10.3390/ijms23116148 (PMC9181813; doi:10.3390/ijms23116148)
Supplement: Supplementary file 1 [file ijms-23-06148-s001.zip › ijms-1737077-supplementary/Supplementary material.pdf]

Table S1. Variance analysis of shoot length (SL), root length (RL), shoot weight (SW), and root weight (RW) under Cd treatments with different stress concentration (0, 5, 15, 30, and 60 μM Cd) and time (0, 6 h, 24 h, 3 d, 7 d, and 14 d).

**SV**	**SL**		**MRL**		**SW**		**RW**	
	**F**	**Sig.**	**F**	**Sig.**	**F**	**Sig.**	**F**	**Sig.**
ST	164.5	**	91.6	**	536.9	**	159.5	**
C	13.2	**	10.3	**	187.6	**	42.7	**
V	2.9	ns	16.0	**	0.1	ns	111.4	**
ST × C	32.1	**	11.2	**	151.5	**	35.6	**
ST × V	1.3	ns	0.2	ns	3.1	*	17.2	**
C × V	6.9	**	5.8	**	21.8	**	44.5	**
ST × C × V	2.6	**	0.6	ns	3.9	**	17.0	**

Note: SV represent source of variance. ST, C, and V represent stress time, Cd concentration, and variety, respectively. ST × C, ST ×V, C × V, and ST × C × V represent the interaction of Time and Cd concentration, Time and variety, Cd concentration and variety, and Time, Cd concentration and variety, respectively. * and ** represent significant effect at $p < 0.05$ and $p < 0.01$ level, respectively; and ns represents the effect was not significant.

Table S2. Summary of read generated, quality control and mapping rates of each sample.

Sample	Raw reads	Clean reads	Error rate%	Q20%	Q30%	GC content%	Total mapped	Map rate%
SH0_1	52679032	52038206	0.023	98.6	95.8	52.9	43427053	83.5
SH0_2	56498734	55916014	0.023	98.7	95.9	52.3	45867271	82.0
SH0_3	56815830	56145654	0.023	98.7	96.0	52.2	45969258	81.9
SH1_1	63159492	62631116	0.024	98.5	95.7	54.6	57346488	91.6
SH1_2	63594812	63055858	0.023	98.7	96.1	54.4	58531126	92.8
SH1_3	62974714	62384678	0.023	98.7	96.0	54.0	57665377	92.4
SH2_1	61085426	60541024	0.023	98.6	95.9	54.2	55519553	91.7
SH2_2	62696062	62109544	0.023	98.6	96.0	53.4	56586831	91.1
SH2_3	65966108	65362954	0.024	98.6	95.7	54.5	60583636	92.7
SH3_1	60205910	59642372	0.023	98.7	95.9	54.2	56513013	94.8
SH3_2	59635490	59044662	0.023	98.7	96.0	54.4	55833650	94.6
SH3_3	60916570	60386114	0.023	98.6	96.0	54.5	57240454	94.8
SH4_1	53237410	52718390	0.023	98.8	96.3	54.1	48490836	92.0
SH4_2	52776988	52303916	0.023	98.8	96.3	53.8	50094425	95.8
SH4_3	58009646	57471052	0.023	98.8	96.3	54.4	53251639	92.7
SH5_1	60754912	60161220	0.023	98.7	95.9	54.5	55254932	91.8
SH5_2	59052876	58445334	0.023	98.8	96.1	54.1	54441483	93.2
SH5_3	65633304	64982442	0.023	98.8	96.2	53.8	59933131	92.2
TL0_1	57880746	57238316	0.024	98.4	95.3	53.5	51924133	90.7
TL0_2	51247228	50582834	0.023	98.6	95.8	52.6	45377929	89.7
TL0_3	53515050	52940774	0.024	98.5	95.6	53.1	48241551	91.1
TL1_1	61856924	61266536	0.024	98.4	95.5	53.8	55896790	91.2
TL1_2	66496644	65928702	0.024	98.5	95.6	54.0	60473478	91.7
TL1_3	68355592	67748828	0.023	98.6	95.8	54.4	63875937	94.3
TL2_1	59059340	58493728	0.024	98.4	95.5	54.9	53468487	91.4
TL2_2	62212470	61540882	0.023	98.6	95.8	54.2	56852477	92.4
TL2_3	61238432	60701828	0.024	98.5	95.7	54.2	55891466	92.1
TL3_1	66572444	65980570	0.023	98.7	96.0	54.2	63155526	95.7
TL3_2	61693176	61042496	0.025	98.1	94.7	53.7	57951116	94.9
TL3_3	63170678	62606564	0.024	98.5	95.7	53.8	59348002	94.8
TL4_1	56677640	56127888	0.024	98.6	95.8	54.5	53246539	94.9
TL4_2	62627556	61935646	0.025	98.1	94.8	54.0	57783474	93.3
TL4_3	69514316	68831266	0.023	98.6	95.9	54.2	65218267	94.8
TL5_1	57855498	57040590	0.025	98.2	94.7	53.1	54264156	95.1
TL5_2	62994668	62338208	0.023	98.8	96.1	53.9	58980159	94.6
TL5_3	48872366	48281830	0.023	98.7	96.0	53.4	45813702	94.9
Min	48872366	48281830	0.023	98.1	94.7	52.2	43427053	81.9
Max	69514316	68831266	0.025	98.8	96.3	54.9	65218267	95.8
Mean	59902877	59304050	0.023	98.6	95.8	53.8	54695686	91.9

Table S4. Primer sequences used for qRT-PCR.

Serial number	Gene_id	Forward primer	Reverse primer
Actin	*18S*	CGTCGCGTCCACCCTTTG	GATTTGAAGGTTCCAACTTTG
G1	*longmi034279*	ATGCATCGGTGCTTGACGGA	CAGATGGCTTCATCAGAGCTGC
G2	*longmi049466*	ACCATGAAGAACCCCACCGA	ACAATGTCCTGGTCGCTCAA
G3	*longmi063515*	TCCTGCACCTTTGGAGGATATG	GGATACGTCGGAGCCTGTTA
G4	*longmi015908*	GGTCCCATCACCAAAGTGCT	TTGGCGTTGATGAGCAGAGG
G5	*longmi050998*	ACTGTTGTCCTCAAGGTCGCC	CCCGACTTGGAAACCGTCT
G6	*longmi010223*	CAGCCTGCTGATACACACCA	CTGAACTGGCTCAGCTGCAA
G7	*longmi057601*	CGGCAAGTGCTATTCAGTGG	GCTCAGCCAAACCAGATCCT
G8	*longmi023804*	TCCTTTCTCCACTATCACGCC	GGTTGGCAGGACCACAGTTA
G9	*longmi011218*	CAACCGACGATACCGACACG	TGGTTCCTCACCTACGAGCG
G10	*longmi040957*	ACAGATAGACGTGCGGAATCAG	ACTGCTTCAGGCCCAACAAC

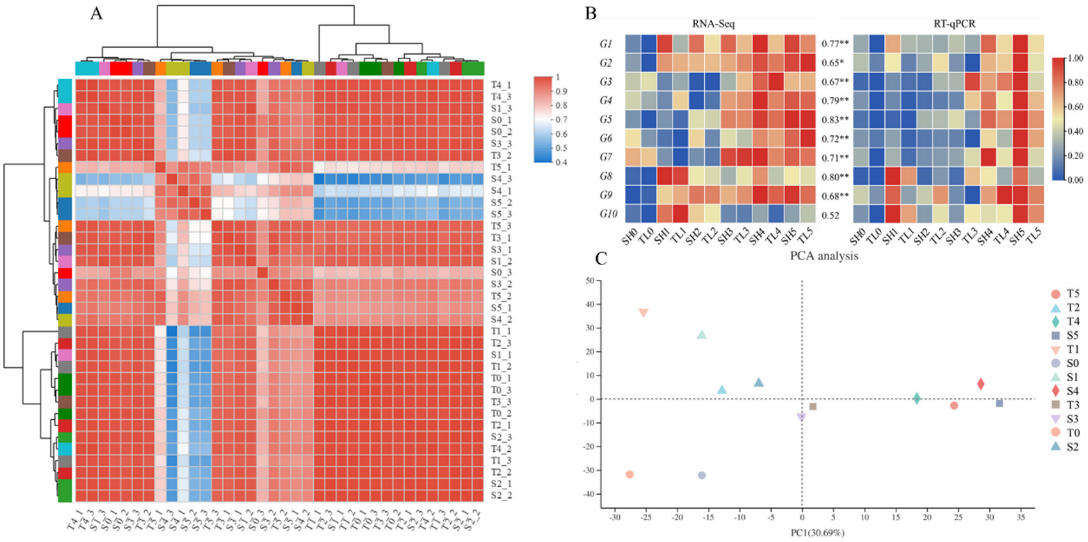

Figure S1. Correlation analysis between samples **(A)**, qRT-PCR validation of expression profiles of DEGs **(B)**, and principal component analysis (PCA) **(C)**.
